# Immature neutrophils and myeloid-derived suppressor cells in sepsis: differences in occurrence kinetics

**DOI:** 10.1186/s13054-023-04781-3

**Published:** 2024-01-02

**Authors:** Rémy Coudereau, Muzhda Haem Rahimi, Anne-Claire Lukaszewicz, Martin Cour, Frank Bidar, Laurent Argaud, Fabienne Venet, Guillaume Monneret

**Affiliations:** 1 Immunology Laboratory, Edouard Herriot Hospital, Hospices Civils de Lyon, 5 Place d’Arsonval, 69437 Lyon Cedex 03, France; 2 EA 7426 “Pathophysiology of Injury-Induced Immunosuppression”, Université Claude Bernard Lyon 1-Hospices Civils de Lyon-bioMérieux, Lyon, France; 3 Anesthesia and Critical Care Medicine Department, Edouard Herriot Hospital, Hospices Civils de Lyon, Lyon, France; 4 Medical Intensive Care Department, Edouard Herriot Hospital, Hospices Civils de Lyon, Lyon, France; 5 CIRI, Centre International de Recherche en Infectiologie, Univ Lyon, Inserm, U1111, CNRS, UMR5308, ENS de Lyon, Université Claude Bernard Lyon 1, 69007 Lyon, France

**To the Editor,**

The disease course of sepsis is complex and multifaceted, involving both hyperinflammation (leading to disruption of homeostasis and organ failure) and counter anti-inflammatory feedback mechanisms aimed at limiting deleterious inflammation. In a significant number of patients, the latter contributes to the development of pronounced immunosuppression, the extent and duration of which are associated with a poor outcome.

Among the factors contributing to this sepsis-induced immunosuppression, clinical observations [1, 2] have highlighted the pivotal role of the delayed emergence of myeloid-derived suppressor cells (MDSCs), a heterogeneous population of immature myeloid cells known for their immune suppressive activity. However, conducting routine whole blood monitoring of these cells remains challenging for practical clinical application. On the one hand, MDSCs derived from the monocytic lineage can be readily identified by the presence of CD14 and the decrease in HLA-DR molecules [2, 3]. On the other hand, MDSCs originating from the neutrophilic lineage are identified by a nonspecific immature phenotype, their low-density post-Ficoll purification, alongside their immunosuppressive functions that need be demonstrated in vitro [3]. Therefore, in whole blood, discerning genuine MDSCs originating from the granulocytic lineage from immature neutrophils (IN) like CD10^low^CD16^low^ neutrophils formed during emergency granulopoiesis but lacking suppressive properties, poses a challenge due to their overlapping immature phenotypic traits. Recently, LOX-1 has been proposed as a specific marker for MDSCs, which could aid in resolving this diagnostic challenge. Initially described in cancer patients [4], the presence of LOX-1 MDSC has also been observed in sepsis [5]. We leveraged this advancement in phenotyping to simultaneously assess IN and LOX-1 MDSC in both bacterial and viral sepsis over the 1st week of stay in ICU.

Fifteen COVID-19 ICU patients and 17 patients with bacterial septic shock sampled 3 times during the 1st week after ICU admission (D0, D3, and D7) were included. Main clinical characteristics are presented in Fig. 1A. Firstly, we observed a significantly increased percentage of IN in septic shock patients upon ICU admission (Fig. 1B) which likely reflects the emergency hematopoiesis triggered by the massive production of inflammatory cytokines observed in septic shock. This elevation was not observed in COVID-19 patients probably because of the lower initial severity (as indicated by SAPSII and SOFA scores). In contrast, we observed a parallel, progressive increase in LOX-1 MDSCs in both types of sepsis, with a similar peak being reached at the end of the 1st week (Fig. 1B). This confirmed previous observations describing MDSC occurrence as a delayed process after sepsis. This indicates that the phenotype of IN is not relevant to identify MDSC during the first few days after septic shock. Secondly, in COVID-19 patients, the percentages of IN and LOX-1 MDSCs were significantly correlated (*r* = 0.72, *p* < 0.001) when considering all samples over time (Fig. 1C), and a noticeable trend was observed at D7 (*r* = 0.83, *p* = 0.058). In sharp contrast, this correlation was entirely absent in septic shock patients when considering all samples over time, but it became positive at D7. This highlights that in sepsis at D0, the vast quantity of IN are LOX-1 negative, whereas on D7, the LOX-1 positive cells exhibit an immature phenotype (Fig. 1D).

**Fig. 1:**
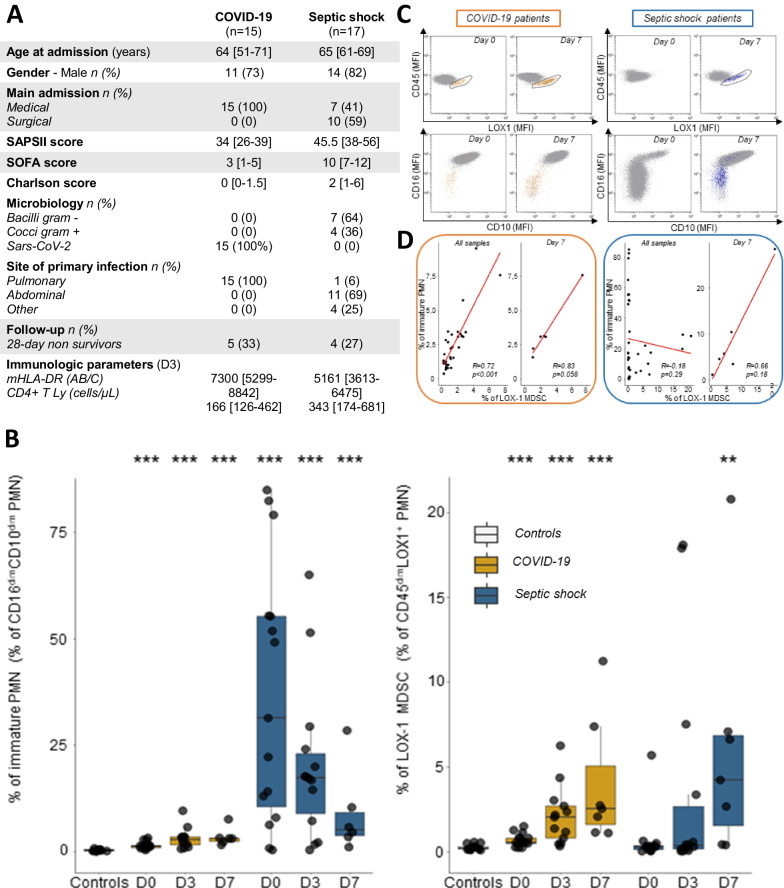
Monitoring of immature neutrophils and LOX‐1 PMN‐MDSC over time in COVID-19 and septic shock patients. **A** Table depicting patients’ main characteristics. **B** Percentages of immature neutrophils (CD10^low^CD16^low^) among total neutrophils were measured in peripheral blood from 15 healthy controls and 14 COVID‐19 ICU patients (D0: n = 14; D3: n = 12; and D7: n = 6), or 17 septic shock patients (D0: n = 15; D3: n = 14; and D7: n = 6). Percentages of LOX‐1 MDSCs among total neutrophils were measured in peripheral blood from 15 healthy controls and 15 COVID‐19 ICU patients (D0: n = 15; D3: n = 13; and D7: n = 7), or 17 septic shock patients (D0: n = 14; D3: n = 13; and D7: n = 7). Nonparametric Mann–Whitney U-test was used to compare values between controls and patients (** *p* < 0.01 and *** *p* < 0.001). **C** Correlation of LOX‐1 MDSCs and immature neutrophils in COVID-19 patients (left panel) and septic shock patients (right panel). Correlations were calculated using Spearman correlation test. **D** Representative examples (D0 and D7) of LOX‐1 MDSCs (CD45^low^LOX-1^pos^, supper panel) and immature neutrophils (CD10^low^CD16^low^, lower panel) characterization for one COVID‐19 ICU patient (left panels) and one septic shock patient (right panels). Cells were gated on whole neutrophil population based on CD45/SSC characteristics. LOX-1 MDSCs from COVID-19 patients are coloured in orange. LOX-1 MDSCs from septic shock patients are coloured in blue

The differences in the appearance of IN and MDSC in these two sepsis contexts can be attributed to several factors. Among them, the magnitude of the inflammatory response is likely of major importance. In contrast with septic shock, COVID-19, even in its most severe presentation, involves a progressive deterioration lasting for 5–7 days before ICU admission. This evolution is accompanied by a low-grade inflammation, which is much lower in intensity compared to that observed in septic shock and could be also attenuated by dexamethasone. As a result, it does not trigger a massive emergency granulopoiesis. Furthermore, as a viral infection, COVID-19 primarily involves antiviral processes that do not require neutrophils as the first line of defence. Alongside the variation in IN, the similarities in LOX-1 MDSCs kinetics between COVID-19 and septic shock illustrate that, once organ failures appear in severe infections, common mechanisms of immunosuppression occur regardless of the underlying cause, in a delayed fashion. This observation is in accordance with Hollen's findings [1], which showed that the acquisition of suppressive functions by MDSCs typically takes approximately 1 week.

To the best of our knowledge, this is the first sepsis report that involves the simultaneous assessment of IN and MDSCs. As such, it warrants further confirmation in larger cohorts of patients. That given, as of now, our study underscores the significance of accurately defining the MDSCs phenotype in whole blood for future sepsis studies. Mere reliance on the immature phenotype (such as CD10^low^CD16^low^) appears to be insufficient for identifying these cells, especially in the early days following sepsis onset, without functional testing to establish their immunosuppressive properties.

## Data Availability

The datasets used and/or analysed during the current study are available from the corresponding author on reasonable request.

## Abbreviations

MDSCs: Myeloid-derived suppressor cells
IN: Immature neutrophils
ICU: Intensive care unit
LOX-1: Lectin‐type oxidized LDL receptor 1
